# Complete genome sequence data of Priestia megaterium strain MARUCO02 isolated from marine mangrove-inhabited sediments of the Indian Ocean in the Bagamoyo Coast

**DOI:** 10.1016/j.dib.2023.109119

**Published:** 2023-04-07

**Authors:** Reuben S. Maghembe, France P. Mdoe, Abdalah Makaranga, James A. Mpemba, Deogratius Mark, Clement Mlay, Edward A. Moto, Andrew G. Mtewa

**Affiliations:** a Biological and Marine Sciences, Faculty of Natural and Applied Sciences, Marian University College, P. O. Box 47, Bagamoyo, Tanzania; b Department of Biological Sciences, Faculty of Science, University of Botswana, Private Bag 0704, Gaborone, Botswana; c Department of Biochemistry and Physiology, St Francis University College of Health and Allied Sciences, P.O.Box 175, Ifakara, Tanzania; d Tanzania Agricultural Research Institute, Mikocheni, P.O. Box 6226, Dar es Salaam, Tanzania; e Biosciences Research Centre (PUBReC), Pwani University, Kenya; f Department of Biology, College of Natural & Mathematics Sciences, University of Dodoma, P.O Box 338, Dodoma, Tanzania.; g Chemistry Section, Department of Applied Sciences, Malawi Institute of Technology, Malawi University of Science & Technology, P.O. Box 5196, Limbe, Malawi

**Keywords:** Priestia megaterium, Genome sequence, Phylogenomics, Biosynthetic gene clusters, Carotenoids, Polyhydroxyalkanoates, MARUCO02

## Abstract

*Priestia* is a genus of biotechnologically important bacteria adapted to thrive in a wide range of environmental conditions including the marine sediments. Here, we screened and isolated a strain from the Bagamoyo marine mangrove-inhabited sediments and then employed whole genome sequencing to recover and define its full genome. *De novo*-assembly with Unicycler (v. 0.4.8) and annotation with Prokaryotic Genome Annotation Pipeline (PGAP) revealed that that its genome contains one chromosome (5,549,131 bp), with a GC content of 37.62%. Further analysis showed that the genome contains 5,687 coding sequences (CDS), 4 rRNAs, 84 tRNAs, 12 ncRNAs, and at least 2 plasmids (1,142 bp and 6,490 bp). On the other hand, antiSMASH-based secondary metabolite analysis revealed that the novel strain (MARUCO02) contains gene clusters for biosynthesis of MEP-DOXP-dependent versatile isoprenoids (eg. carotenoids), siderophores (synechobactin and schizokinen) and polyhydroxyalkanoates (PHA). The genome dataset also informs about the presence genes encoding enzymes required for generation of hopanoids, compounds that confer adaption to harsh environmental conditions including industrial cultivation recipes. Our data from this novel *Priestia megaterium* strain MARUCO02 can be used for reference and in genome-guided selection of strains for production of isoprenoids as well as industrially useful siderophores and polymers, amenable for biosynthetic manipulations in a biotechnological process.

**Specifications Table**

**Table:** 

Subject	*Omics: Genomics, Biochemistry and Molecular Biology*
Specific subject area	*Marine bacterial genomics*
Type of data	• Raw sequence reads • DNA sequences in FASTA format • Tables • Figures.
How the data were acquired	Sequence reads were generated via Illumina Novaseq 6000 whole genome sequencing. Then reads were quality-controlled by FastQC and Trimmomatic, assembled with Unicycler and then annotated with PGAP. The phylogram was generated with TYGS and the biosynthetic pathways were manually curated following PGAP annotation, antiSMASH and PRISM predictions.
Data format	Raw, analyzed and assembled DNA sequences
Description of data collection	Whole genome sequencing, assembly and annotation
Data source location	• *Marian University College* • *Bagamoyo* • *Tanzania*
Data accessibility	Repository name: NCBI GenBank Data identification number: SRA data: PRJNA887360, Nucleotide accession numbers CP107543.1, CP107541.1, and CP107540, with BioSample and BioProject numbers SAMN31165207 and PRJNA887360 respectively. Direct URL to data: https://www.ncbi.nlm.nih.gov/sra/PRJNA887360 https://www.ncbi.nlm.nih.gov/bioproject/887360 https://www.ncbi.nlm.nih.gov/biosample/?term=SAMN31165207 https://www.ncbi.nlm.nih.gov/nuccore/NZ_CP107543.1 https://www.ncbi.nlm.nih.gov/nuccore/NZ_CP107542.1 https://www.ncbi.nlm.nih.gov/nuccore/NZ_CP107541.1 https://www.ncbi.nlm.nih.gov/assembly/GCA_025837055.1

## Value of the Data

• The genome data for *Priestia megaterium* strain MARUCO02 could present a potential strain for study of industrial production of enzymes, siderophores, polymers and isoprenoid compounds such as carotenoids.
• The genome data can benefit scientific innovation in the laboratory and industrial setting.
• By means of both raw and analyzed datasets, the genome dataset possesses value for comparative genomic studies characterizing marine plant-associated and growth promoting *Priestia* species.

## 1 Objective

*Priestia megaterium* has emerged as a bacterial species with application in the biotechnological industry, with its role as source of enzymes, vitamins, pigments and polymers. This study sought to recover novel strains of *Priestia megaterium* from local marine ecosystems for biotechnological applications. We thus aimed at uncovering the genome-guided biotechnological relevance of *Priestia megaterium* strain MARUCO02 isolated from Bagamoyo Tanzania.

## 2 Data Description

The dataset in the current article describes the genomic features underlying the biotechnological potential of *Priestia megaterium* strain MARUCO02 as a source of various high value bioproducts. A total of 16,631,008 paired end reads (raw data) were generated, which upon filtration were reduced to 10,429,051. *De novo* assembly resulted in 39 contigs, with N50 of 4283054, and a total of 5,614,752 bp. Shown with genome features in Table 1, the resolved chromosome has the size of 5,549,131 bp with a total of 5.592 coding sequences (CDS) and 88 RNAs. Table 2 describes the average nucleotide identity (ANI) values showing the possible closest relatives of the MARUCO02 strain, useful for future comparative studies.

**Table 1:** Genome features of *Priestia megaterium* strain MARUCO02. Gene features are a result of combined output from PGAP and PATRIC annotation.

Features	Chromosome	Plasmid 2	Plasmid 4
Genome size (bp)	5,549,131	1,142	6,490 bp
GC content (%)	37.62	55,08	39.97
CDs	5,592	1	7
tRNAs	84	0	0
rRNAs	4	1	2
Genes assigned to GO	1,039	0	0

**Table 2:** Selected closest strains identified from PATRIC annotation and BLASTn of the 16S rRNA gene. Genome sizes for the strains are shown to match the possible size with the MARUCO02 strain. The selected genomes were retrieved from the NCBI genome and assembly databases. The ANI values were computed to compare the MARUCO02 strain with each close relative in the table.

Strain	Genome size	Genome/Assembly accession number	Genus	Species	ANI (%)
MARUCO02	5,549,131	CP107543.1	*Priestia*	*P. megaterium*	100
DSM 319	5,097,447	CP001982.1	*Priestia*	*P. megaterium*	96.22
QM B1551	5,523,192	CP001983.1	*Priestia*	*P. megaterium*	95.94
NBRC 15308 = ATCC 14581	5,746,640	CP009920.1	*Priestia*	*P. megaterium*	95.95
NCT-2	5,883,957	CP032527.2	*Priestia*	*P. megaterium*	95.98
B8W22	5,095,483	GCF_000956595.1	*Priestia*	*P. aryabhattai*	97.74

**Fig 1:**
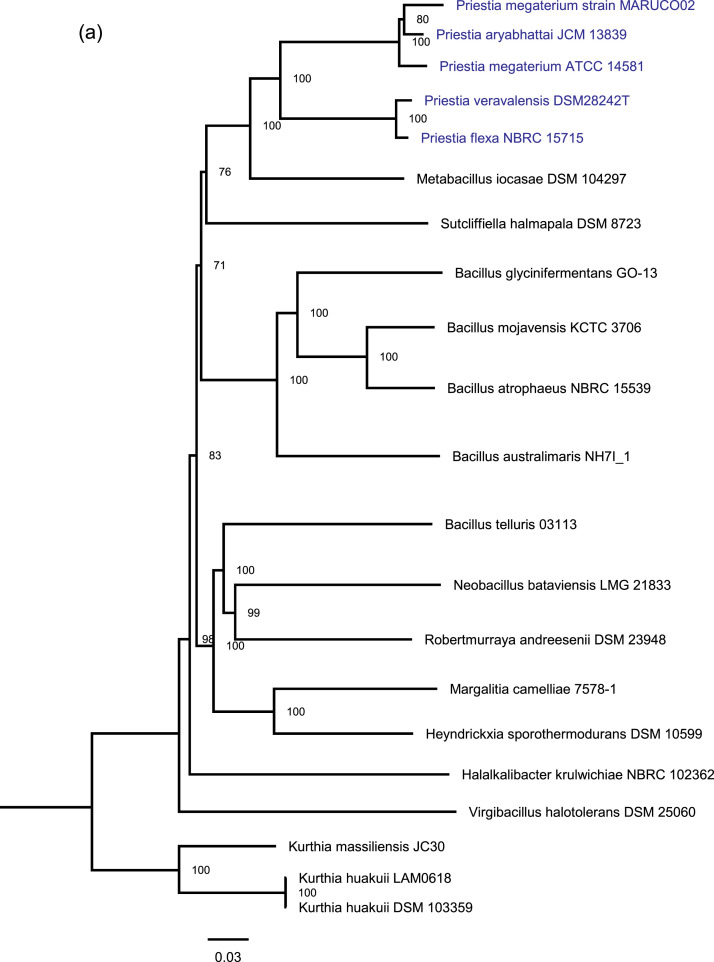

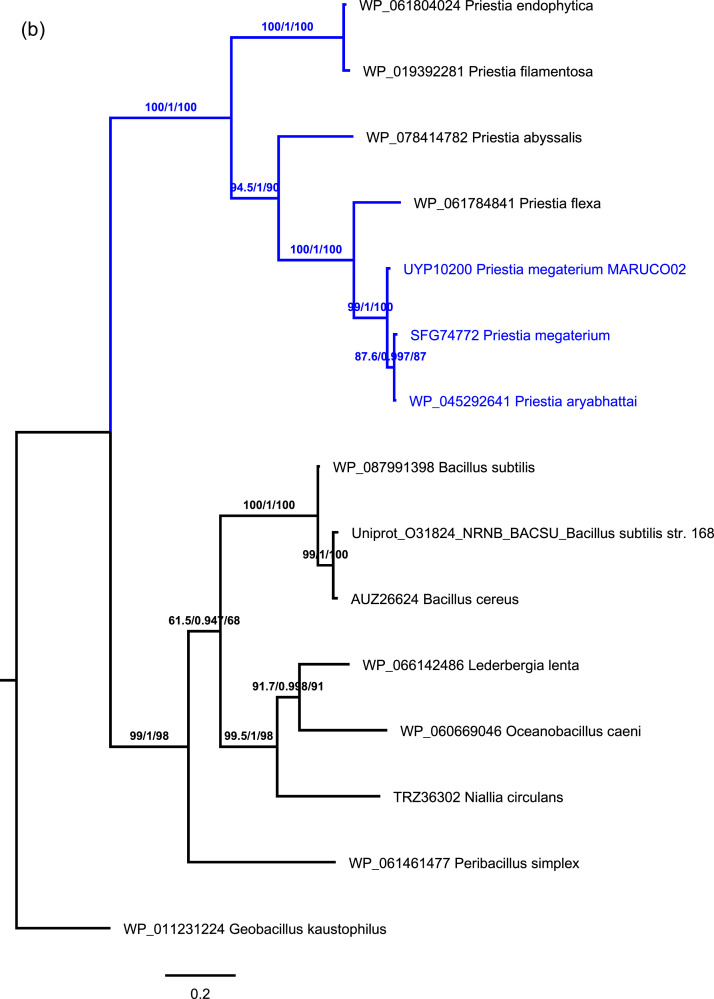
(a) A proteome-based placement of *Priestia megaterium* MARUCO02 inferred with FastME 2.1.6.1 [9] from GBDP distances (branch length formula d5). The position of the MARUCO02 strain is highlighted in blue. Branch values are GBDP pseudo-bootstrap support values > 60 % from 100 replications, with an average branch support of 93.8% The tree was rooted at the midpoint [10]. (b) Phylogenetic position of the MARUCO02 strain inferred with Maximum Likelihood (ML) method and Bayesian estimation from the LG substitution model for the NrnB DHHA1 domain-containing protein. The blue color is applicable to indicate the clade under which the MARUCO02 strain falls.Numbers on each node represent the bootstrap values from both ML and Bayesian estimation from 1000 replicates. The tree is rooted at the midpoint, with *Geobacillus kaustophilus* as an outgroup.

Fig. 1 represents the phylogenomic position (a) of *Priestia megaterium* strain MARUCO02 based on whole whole-proteome GBDP distances generated using TYGS [1] and the conserved DHHA1 domain-containing protein phylogeny (b) from maximum likelihood inferred based on the LG substitution model using IQ-TREE [2]. From various previous historical studies *Bacillus* and *Priestia* were placed together in the same genus as *Bacillus*. Until recently, genomic studies have unanimously broken the genus *Bacillus* into multiple taxonomic groups, guiding the placement *Priestia* into a separate genus [3], [4], [5]. This genome dataset places our strain in the genus *Priestia* and species *Priestia megaterium*.

**Fig 2:**
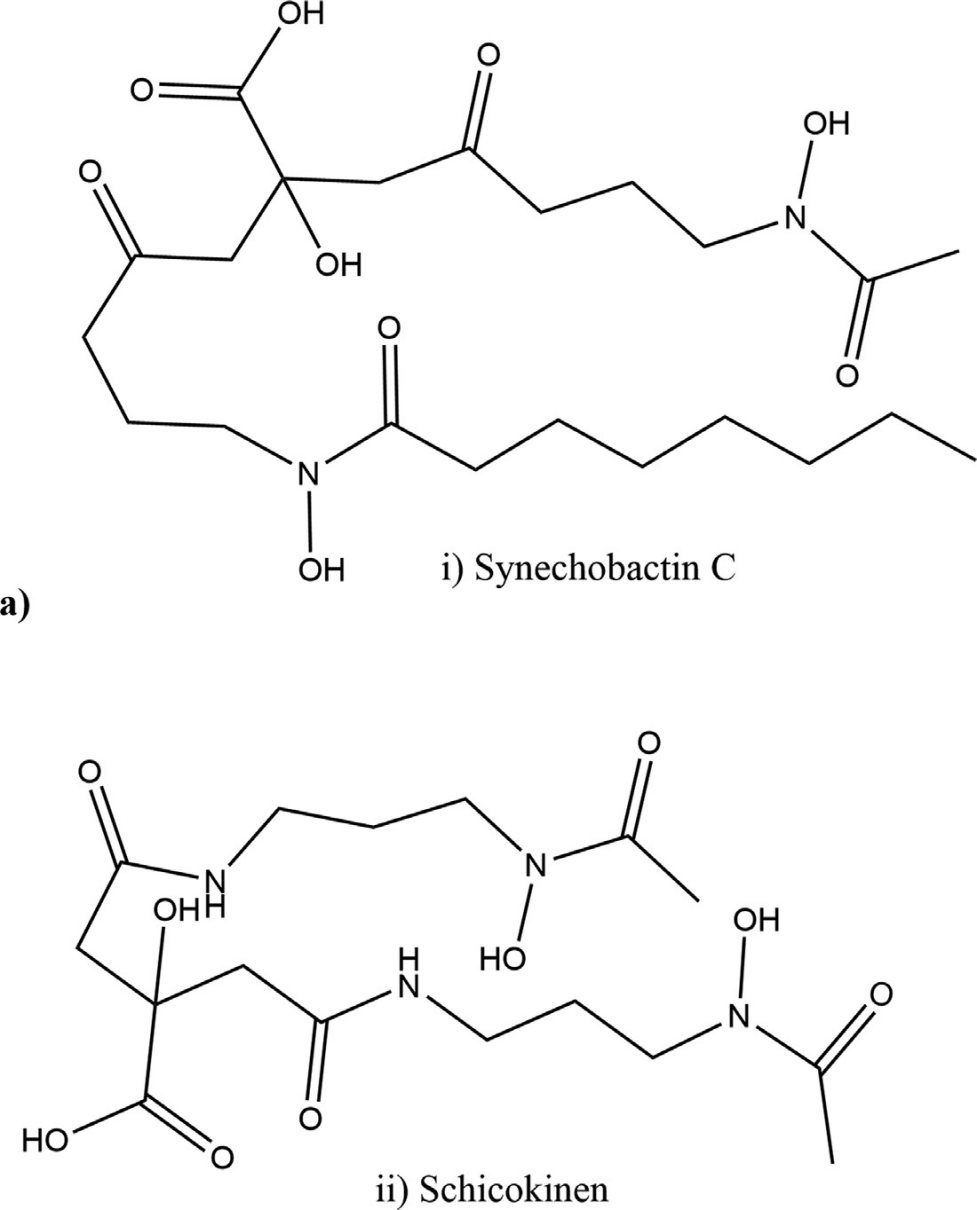

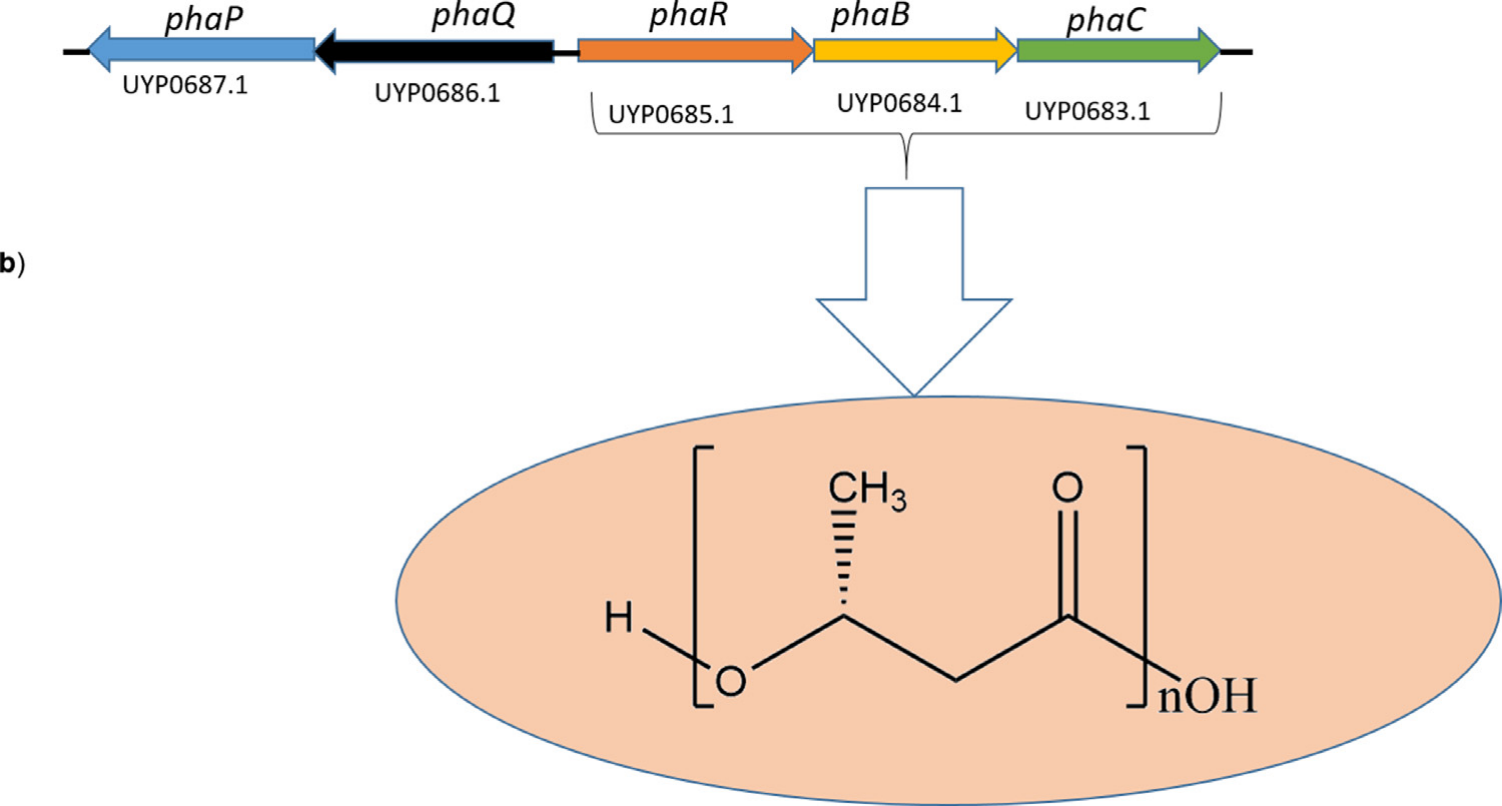
(a) Structures of siderophores predicted from antiSMASH analysis and drawn with Chemdraw, i.e. synechobactin and schizokinen. The two structures were drawn using Chemdraw. b) The PHA biosynthetic gene cluster decoded from genome annotation of P. megaterium strain MARUCO02. The cluster comprises of two regulatory genes (PhaP and PhaQ) and structural genes (PhaR, PhaB and PhaC) responsible for PHA polymer assembly.

To delineate the biosynthetic potential for secondary metabolites with antiSMASH the genome was broken into seven regions (Supplementary file 2). Of all the seven regions, only two (region 1 and region 7) significantly matched known biosynthetic clusters. Region 1 carries three significant cluster hits, i.e. BGC0002470, BGC0002633 and BGC0002683, which correspond to structurally related siderophores namely synechobactin and schizokinen (Fig. 2(a)). Region 7 represents one significant hit cluster BGC0000645, which belongs to the carotenoid class of compounds.

Whereas antiSMASH does not predict structural assembly of respective compounds, PRISM commonly does. However, in this work, although PRISM could not resolve any structure, we were able to confirm one cluster, the siderophore, with the core open reading frames (ORFs), supporting the BGCs predicted from antiSMASH.

Fig. 2(b) represents the gene cluster for biosynthesis of polyhydroxyalkanoates (PHAs). The dataset shows the gene cluster and enzymes that responsible for catalytic steps from initial reactions to assembly of the monomers into polymers.

**Fig 3:**
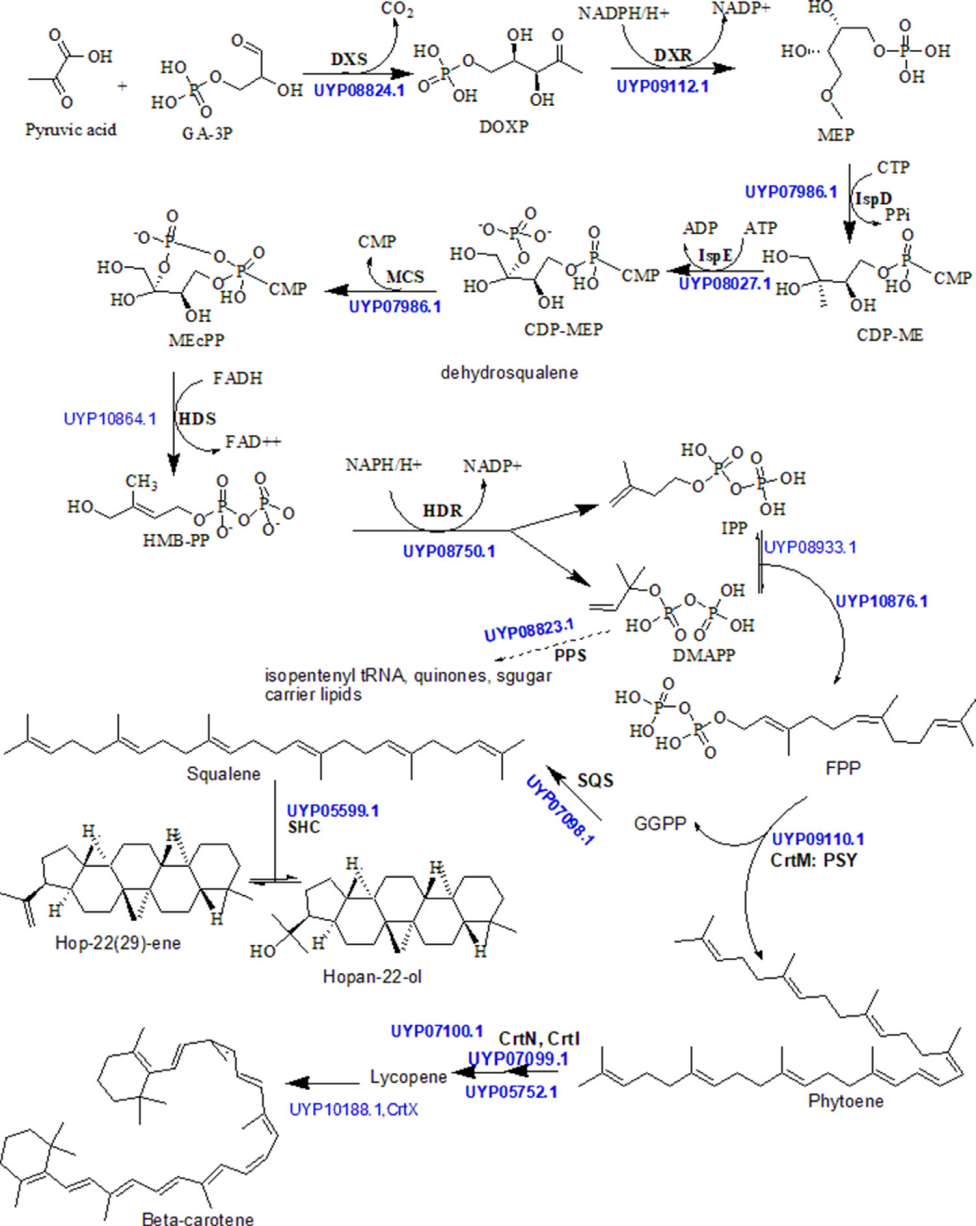
The carotenoid biosynthetic pathway for P. megaterium strain MARUCO02 genome. The pathway was reconstructed by combining antiSMASH BGC analysis with manual protein functional annotation and confirmation with databases (NCBI (https://blast.ncbi.nlm.nih.gov/Blastp) and UniProt (https://www.uniprot.org/blast)). Genes encoding the respective enzymes are represented in black bolded letters together with the accession numbers (blue bolded) of individual protein sequences manually sorted from PGAP annotation.

Fig. 3 represents the non-mevalonic acid pathway, also known as the 2-C-methyl-D-erythritol 4-phosphate/1-deoxy-D-xylulose 5-phosphate (MEP/DOXP) pathway for isoprenoid biosynthesis, predicted form antiSMASH and then recovered via manual curation of the PGAP genome annotation dataset. Chemical structures were drawn using Chemdraw 8.0 [6], along with all enzymes catalyzing important reactions.

### 2.1 Taxonomy and phylogenetic placement

We combined PATRIC, TYGS and PGAP annotation results to infer the taxonomic and phylogenetic position of the MARUCO02 strain. From PATRIC, the genome was annotated using the RAST tool kit (RASTtk) [7]. The annotation results (Table 2) confirmed that strain belongs to the genus *Priestia.* The closest reference and representative genomes to were identified by Mash/MinHash [8]. The closest relatives belong to the species *Priestia megaterium* and *Priestia aryabhattai.* However, detailed analysis indicates *P. aryabhattai* still matches with *P. megaterium* with average nucleotide identity (ANI) of above the threshold (95%). Thus the two species are suggestively a single species with variable strains. Genomes of *Priestia species* possess unique oligoribonuclease NrnB or cAMP/cGMP phosphodiesterase and DHH/DHHA1 superfamily protein, which have been utilized as reliable molecular signatures distinguishing the genus from the rest [4]. We manually searched the protein sequences from the PGAP- recovered proteome and hereby confirm the presence of these genes (UYP05222.1, UYP05090.1, UYP08660.1 and UYP10200.1) in our strain. Guided by the recent taxonomic demarcation by Gupta et al. [4], we aligned the UYP10200.1 protein with those from *Priestia* and *Bacillus* clades, from which the phylogenetic tree generated by IQ-TREE confirmed that the MARUCO02 falls under the genus *Priestia*, together with *P. megaterium* and *P. aryabhattai*, among other strains (Fig. 1(b)).

Based on ANI, TYGS and IQ-TREE phylogenies, we unanimously named this species *Priestia megaterium*. In all the data the ‘’MARUCO2’’ strain identifier signifies isolation and handling by Marian University College (MARUCO) as a second isolate (02) from our project.

### 2.2 Biosynthesis of Secondary Metabolites of Interest

Analysis with PRISM recovered only one cluster, which was identified as a siderophore biosynthetic cluster. The cluster contains three core open reading frames (ORFs), identified as the iron-binding IucA/IucC family siderophore biosynthesis protein (WP_182005752.1), thymidylate synthase (WP_182005751.1), and dihydrofolate reductase (WP_182005752.1). From PGAP annotation and BLASTp corresponding output, the BGC could not reveal a clear compound from PRISM until the antiSMASH data were comparatively used (Supplementary file 2). From antiSMASH prediction, there are three possible BGCs for two closely related siderophores, namely synechobactin and schizokinen (Fig. 2a). The latter was described for the first time in *Priestia megaterium* in early 1970s as an iron-transporting molecule [11]. While most of the siderophores are known to be synthesized via the nonribosomal peptide synthetase (NRPS) pathway, synechobactin and schizokinen are generated by a NRPS-independent siderophore synthetase (NIS) pathway and are well characterized in Cyanobacteria [12]. Here, through the combination of PRISM and antiSMASH BGC prediction, we highlight the potential engagement of *Priestia megaterium* MARUCO02 in the biosynthesis of these siderophores, useful in bioremediation as well as medicine [12].

In addition, the MARUCO02 genome contains BGCs for polyhydroxyalkanoate synthesis (Fig. 2b) as well as possible degradative genes. The gene cluster for PHA has been well characterized in *Priestia megaterium* about two decades ago [13,14]. The cluster consists of an operon with *PhaP, PhaQ, PhaR, PhaB* and *PhaC*. Although the cluster could not be unraveled with antiSMASH or PRISM, we manually searched each candidate gene from the proteome recovered from PGAP annotation, and we hereby present them in Fig. 2(b). While the two upstream genes *PhaP*, and *PhaP* comprise a regulatory unit, the three *PhaR, PhaB* and *PhaC* are responsible for generation of PHA units and their polymerization to complete PHA molecules [14]. Interestingly, we were also able to identify the polyhydroxybutyrate (PHB) depolymerase gene from PGAP annotation (NCBI accession UYP05899.1). The enzyme PHB depolymerase (EC 3.1.1.75) is of interest in biodegradation research [15,16], thus the MARUCO02 genome is suggestive of the potential as factory for enzymes required in biodegrading of polymers including plastics.

In the biosynthesis of terpenoids, the MARUCO02 genome possesses genes for MEP DOXP pathway responsible for biosynthesis of an array of carotenoids. Primarily recovered from antiSMASH known cluster blast algorithm [17], the genome was found to exhibit up to 50% similarity with genomes involved in the biosynthesis of carotenoids. Our downstream analysis confirmed the methylerythritol 4-phosphate (MEP)/1-deoxy-D: -xylulose-5-phosphate (DOXP) (DOXP/MEP) pathway with possible versatilities of carotenoids and hopanoids (Fig. 3). Described in multiple reports [18], [19], [20], the MEP/DOXP, also known as the non-mevalonate pathway, is responsible for the biosynthesis of monoterpenes of essential oils, linalyl acetate, several forms of sesquiterpenes, diterpenes, phytol as well as carotenoids. From glycolysis, the enzyme 1-deoxy-D-xylulose 5-phosphate synthase (DXS) condenses a pyruvate molecule with glyceraldehyde-3-phosphate (GA-3P) to form DOXP, which then is reduced to MEP by DOXP reductoisomerase (DXR). The most important rate determining steps include the DXS, squalene synthase (SQS), phytoene synthase (CrtM, PSY) (Fig. 3).

## 3 Experimental Design, Materials and Methods

### 3.1 Strain Isolation and DNA Extraction

Samples were obtained from sediments inhabited by mangrove trees in the Indian Ocean in the vicinity of the sea shore (https://www.google.com/maps/@-6.424511,38.901958,14z) of the Bagamoyo Coast in Tanzania. Three sediment samples were collected using sterile plastic bottles and stored at 4°C in the laboratory. For bacterial isolation a proportion of the sediment (approx 5 g) was dissolved in 200 ml of 0.80% of NaCl followed by serial dilutions (10^−1^ to 10^−6^) with phosphate buffered saline (PBS) (pH 7.2) and isolation by streaking on nutrient agar (NA) culture at 28 °C for 48 hours. One of the sample colonies was chosen for DNA extraction for identification. Total DNA was extracted using a ZymoBIOMICS DNA Miniprep Kit (ZR D4300), based on the manufacturer's guide.

### 3.2 Library Preparation, Genome Sequencing, Quality Control, Assembly and Annotation

Library construction was performed using a TruSeq DNA PCR-Free kit and TruSeq Nano DNA Kit, and whole genome shotgun sequencing was accomplished by the Ilumina Novaseq 6000 platform (2.5G bp), generating short reads of 151 bp on average length. Raw reads were quality-controlled using FastQC and Trimmomatic (v 0.38) based on the following parameters: minimum adapter overlap (stringency): 1 bp, minimum sequence length for both reads before a sequence pair removal: 20 bp. The reads were *de novo*-assembled) into contigs with Unicyler (v. 0.4.8 and a chromosome using CONTIGuator (v.2.7.4) [21] and Medusa (v. 1.6). The chromosome was then annotated with Prokaryotic Genome Annotation Pipeline (PGAP v. 6.3) (https://www.ncbi.nlm.nih.gov/genome/annotation_prok/) using the best-placed reference protein method (set; GeneMarkS-2+). Plasmids were assembled by mapping the contigs with CONTIGuator (v.2.7.4) [21] and Medusa (v. 1.6) to reference *Priestia megaterium* reference plasmids from previous studies [22]. Ribosomal RNA (rRNA) was recovered by scanning the assembled contigs with ContEst16S (https://www.ezbiocloud.net/tools/contest16s) and the probable relative strain was immediately predicted from basic local alignment search tool (BLASTn) against the NCBI database [23].

### 3.3 Taxonomic Placement with Phylogenomic and Phylogenetic Analyses

For whole proteome-based phylogenomic analysis, the genome sequence was uploaded to the Type (Strain) Genome Server (TYGS), a free bioinformatics platform available at https://tygs.dsmz.de [1]. Guided by previous genus delineation [4], the DHHA1, a conserved molecular marker, was chosen and aligned using MAFFT(v7.487) [24] (Supplementary file 1) with those of reference *Priestia* and *Bacillus* strains from the model study by Gupta and colleagues [4]. Phylogenetic inference was deduced from the LG substitution model, with maximum likelihood and Bayesian estimation methods using IQ-TREE (v.16.12) [25] for 1000 replicates.

### 3.4 Analysis of Biosynthetic Gene Clusters and Pathway Elucidation

To predict the clusters and possible structural assembly of secondary metabolites, the genome sequence was scanned with PRISM 4 [26]. Alternatively, the genome was analyzed with antiSMASH (v6.0) [17] based on default parameters to predict the number of clusters that were possibly not resolved by PRISM. Guided by both PRISM and antiSMASH prediction, genes from both BGC analysis tools, also featuring those identified from PGAP annotation, were manually selected and reanalyzed with BLASTp (NCBI (https://blast.ncbi.nlm.nih.gov/Blastp) and UniProt (https://www.uniprot.org/blast)) in order to find more about their relevance to their predicted biosynthetic gene clusters. Structures of the predicted compounds were then drawn using Chemdraw (v8.0) [6] and the pathways were manually curated based on functional annotations of their respective catalytic proteins.

## Ethics Statements

This project did not involve human subjects, animals, cell lines or endangered species. The current manuscript is our original work, which has not been previously published elsewhere.

## CRediT authorship contribution statement

**Reuben S. Maghembe:** Conceptualization, Methodology, Software, Writing – original draft. **France P. Mdoe:** Methodology, Visualization. **Abdalah Makaranga:** Conceptualization, Writing – review & editing, Validation. **James A. Mpemba:** Methodology, Data curation. **Deogratius Mark:** Data curation, Investigation. **Clement Mlay:** Methodology, Investigation. **Edward A. Moto:** Writing – review & editing, Validation, Supervision. **Andrew G. Mtewa:** Writing – review & editing, Supervision.

## Declaration of Competing Interest

The authors declare that they have no known competing financial interests or personal relationships that could have appeared to influence the work reported in this paper.

## Data Availability

Raw, analyzed and assembled DNA sequences (Original data) (NCBI Genbank). Raw, analyzed and assembled DNA sequences (Original data) (NCBI Genbank). Raw, analyzed and assembled DNA sequences (Original data) (NCBI Genbank). Raw, analyzed and assembled DNA sequences (Original data) (NCBI Genbank). Genome data (Original data) (National Center for Biotechnology Information). Raw, analyzed and assembled DNA sequences (Original data) (NCBI Genbank). Raw, analyzed and assembled DNA sequences (Original data) (NCBI Genbank). Raw, analyzed and assembled DNA sequences (Original data) (NCBI Genbank).

## Appendix Supplementary materials

Image, application 1

Image, application 2

## References

[1] Meier-Kolthoff J.P., Göker M. (2019). TYGS is an automated high-throughput platform for state-of-the-art genome-based taxonomy. Nat. Commun..

[2] Trifinopoulos J., Nguyen L.-T., von Haeseler A., Minh B.Q. (2016). W-IQ-TREE: a fast online phylogenetic tool for maximum likelihood analysis. Nucleic Acids Res..

[3] Adiguzel A., Ay H., Baltaci M.O., Akbulut S., Albayrak S., Omeroglu M.A. (2020). Genome-based classification of Calidifontibacillus erzurumensis gen. nov., sp. nov., isolated from a hot spring in Turkey, with reclassification of Bacillus azotoformans as Calidifontibacillus azotoformans comb. nov. and Bacillus oryziterrae as Calidifontibacillus oryziterrae comb. nov. Int. J. Syst. Evol. Microbiol..

[4] Gupta R.S., Patel S., Saini N., Chen S. (2020). Robust demarcation of 17 distinct Bacillus species clades, proposed as novel Bacillaceae genera, by phylogenomics and comparative genomic analyses: description of Robertmurraya kyonggiensis sp. nov. and proposal for an emended genus Bacillus limiting it only to the members of the Subtilis and Cereus clades of species. Int. J. Syst. Evol. Microbiol..

[5] Patel S., Gupta R.S. (2020). A phylogenomic and comparative genomic framework for resolving the polyphyly of the genus Bacillus: Proposal for six new genera of Bacillus species, Peribacillus gen. nov., Cytobacillus gen. nov., Mesobacillus gen. nov., Neobacillus gen. nov., Metabacillus gen. nov. and Alkalihalobacillus gen. nov. Int. J. Syst. Evol. Microbiol..

[6] Mendelsohn L.D. (2004). ChemDraw 8 ultra, windows and macintosh versions. J. Chem. Inf. Comput. Sci..

[7] Brettin T., Davis J.J., Disz T., Edwards R.A., Gerdes S., Olsen G.J., Olson R., Overbeek R., Parrello B., Pusch G.D., Shukla M., Thomason J.A., Stevens R., Vonstein V., Wattam A.R., Xia F. (2015). RASTtk: a modular and extensible implementation of the RAST algorithm for building custom annotation pipelines and annotating batches of genomes. Sci. Rep..

[8] Ondov B.D., Treangen T.J., Melsted P., Mallonee A.B., Bergman N.H., Koren S., Phillippy A.M. (2016). Mash: fast genome and metagenome distance estimation using MinHash. Genome Biol..

[9] Lefort V., Desper R., Gascuel O. (2015). FastME 2.0: a comprehensive, accurate, and fast distance-based phylogeny inference program. Mol. Biol. Evol..

[10] Farris J.S. (1972). Estimating phylogenetic trees from distance matrices. Am. Nat..

[11] Mullis K.B., Pollack J.R., Neilands J.B. (1971). Structure of schizokinen, an iron-transport compound from Bacillus megaterium. Biochemistry.

[12] Årstøl E., Hohmann-Marriott M.F. (2019). Cyanobacterial siderophores-physiology, structure, biosynthesis, and applications. Mar. Drugs.

[13] Lee T.-R., Lin J.-S., Wang S.-S., Shaw G.-C. (2004). PhaQ, a new class of poly-beta-hydroxybutyrate (phb)-responsive repressor, regulates phaQ and phaP (phasin) expression in Bacillus megaterium through interaction with PHB. J. Bacteriol..

[14] McCool G.J., Cannon M.C. (2001). PhaC and PhaR are required for polyhydroxyalkanoic acid synthase activity in Bacillus megaterium. J. Bacteriol..

[15] Hisano T., Kasuya K., Tezuka Y., Ishii N., Kobayashi T., Shiraki M., Oroudjev E., Hansma H., Iwata T., Doi Y., Saito T., Miki K. (2006). The crystal structure of polyhydroxybutyrate depolymerase from penicillium funiculosum provides insights into the recognition and degradation of biopolyesters. J. Mol. Biol..

[16] Sayyed R.Z., Wani S.J., Alarfaj A.A., Syed A., El-Enshasy H.A. (2020). Production, purification and evaluation of biodegradation potential of PHB depolymerase of Stenotrophomonas sp. RZS7. PLoS One.

[17] Blin K., Shaw S., Kloosterman A.M., Charlop-Powers Z., van Wezel G.P., Medema M.H., Weber T. (2021). antiSMASH 6.0: improving cluster detection and comparison capabilities. Nucleic Acids Res..

[18] Furubayashi M., Li L., Katabami A., Saito K., Umeno D. (2014). Construction of carotenoid biosynthetic pathways using squalene synthase. FEBS Lett..

[19] Hunter W.N. (2007). The Non-mevalonate Pathway of Isoprenoid Precursor Biosynthesis *. J. Biol. Chem..

[20] Wanke M., Skorupinska-Tudek K., Swiezewska E. (2001). Isoprenoid biosynthesis via 1-deoxy-D-xylulose 5-phosphate/2-C-methyl-D-erythritol 4-phosphate (DOXP/MEP) pathway. Acta Biochim. Pol..

[21] Galardini M., Mengoni A., Bazzicalupo M. (2015). Mapping contigs using CONTIGuator. Methods Mol. Biol..

[22] Shwed P.S., Crosthwait J., Weedmark K., Hoover E., Dussault F. (2021). Complete genome sequences of priestia megaterium type and clinical strains feature complex plasmid arrays. Microbiol. Resour. Announc..

[23] Morgulis A., Coulouris G., Raytselis Y., Madden T.L., Agarwala R., Schäffer A.A. (2008). Database indexing for production MegaBLAST searches. Bioinformatics.

[24] Katoh K., Rozewicki J., Yamada K.D. (2019). MAFFT online service: multiple sequence alignment, interactive sequence choice and visualization. Briefings Bioinf..

[25] Minh B.Q., Schmidt H.A., Chernomor O., Schrempf D., Woodhams M.D., von Haeseler A., Lanfear R. (2020). IQ-TREE 2: new models and efficient methods for phylogenetic inference in the genomic era. Mol. Biol. Evol..

[26] Skinnider M.A., Johnston C.W., Gunabalasingam M., Merwin N.J., Kieliszek A.M., MacLellan R.J., Li H., Ranieri M.R.M., Webster A.L.H., Cao M.P.T., Pfeifle A., Spencer N., To Q.H., Wallace D.P., Dejong C.A., Magarvey N.A. (2020). Comprehensive prediction of secondary metabolite structure and biological activity from microbial genome sequences. Nat. Commun..

